# Supplementary material for: Perception of sleep duration in adult patients with suspected obstructive sleep apnea
Source: PLoS One. 2020 Aug 27;15(8):e0238083. doi: 10.1371/journal.pone.0238083 (PMC7451567; doi:10.1371/journal.pone.0238083)
Supplement: S1 File — (DOCX) [file pone.0238083.s001.docx]

Percepção do Sono

Favor responder o questionário abaixo após o termino do seu exame:

| - A partir do momento em que você quis dormir, em quanto tempo (minutos) você iniciou o sono? | ________________ minutos |
| --- | --- |
| - Quantas horas você pensa ter dormido durante o exame? | ________________ horas |
| - Quantas vezes você acordou durante o exame? | ­­­­­­­­­­­­­­________________ vezes |
| - Comparado ao número habitual de horas em casa o seu sono no laboratório foi: | Inferior □ Igual □ Superior □ |
| - Você toma diariamente algum medicamento para dormir? | Não □ Sim □ |

Sleep Perception
Please answer the questionnaire below after completing your exam:

| - From the moment you wanted to sleep, how long (minutes) did you start sleeping? | ________________ minutes |
| --- | --- |
| - How many hours do you think you slept during the exam? | ________________ hours |
| - How many times did you wake up during the exam? | ­­­­­­­­­­­­­­________________ times |
| - Compared to the usual number of hours at home, your sleep in the laboratory was: | Lower □ Equal □ Higher □ |
| - Do you take any sleeping pills daily? | No □ Yes □ |
